# Feasibility and acceptability of food‐based complementary feeding recommendations using Trials of Improved Practices among poor families in rural Eastern and Western Uganda

**DOI:** 10.1002/fsn3.964

**Published:** 2019-02-27

**Authors:** Hana Bekele, Florence Turyashemererwa

**Affiliations:** ^1^ World Health Organisation Harare Zimbabwe; ^2^ World Health Organisation Kampala Uganda

**Keywords:** complementary feeding, infant and young child feeding, trails of improved practices, Uganda

## Abstract

Inadequate complementary feeding practices are a major contributor to stunting among children in Uganda. The WHO recommends the promotion of local food‐based complementary feeding recommendations (FBCFRs) to address nutrient gaps during complementary feeding. This study tested the feasibility and acceptability of FBCFRs, using trials of improved practices (TIPs). Qualitative and quantitative methods were used in a cross‐sectional survey over three household visits. At first household visit, information on socio‐demographic factors and food frequency was collected and FBCFRs introduced. The second household visit assessed the use and barriers related to the FBCFRs, while the third household visit assessed the continued use of the FBCFRs. Focus group discussions and key informant interviews provided the insights into community norms on the FBCFRs. Most FBCFRs were feasible and acceptable. However, caretakers found it difficult to implement a full set of FBCFRs together with the recommended frequencies. Caretakers were more likely to try and continue using FBCFRs that had familiar methods of preparation and commonly used ingredients. Seasonality and cost were major barriers to use. Through TIPs, mothers demonstrated that they are open to try new ways of improving their children's nutrition.

## 1 INTRODUCTION

Globally, more than two‐thirds of childhood deaths associated with undernutrition happen in the first year of life and are usually associated with poor infant and young child feeding (IYCF) practices (Jones, Steketee, Black, Bhutta, & Morris, 2003). The IYCF interventions alone avert nearly 20% of child mortality (Bhutta et al., 2008; Jones et al., 2003). The first 1,000 days of a child's life (of which the complementary feeding stage is the longest) are considered a window of opportunity for preventing malnutrition and its associated short‐ and long‐term consequences (Bhutta et al., 2013). The occurrence of undernutrition, characterized by stunting, has been associated with childhood morbidity, premature mortality as well as poor long‐term health, education, and economic outcomes, affecting human potential (Victora et al., 2008; Victora, Onis, Hallal, Blössner, & Shrimpton, 2010).

In Uganda, nearly three out of every ten children (29%) are stunted. The minimum meal frequency and minimum dietary diversity at 45% and 13.0%, respectively, among children are inadequate. The minimum acceptable diet is low (14%). At a regional level, variation in IYCF practices exists with the eastern and western regions of the country having some of the poorest indicators (UBOS, 2016). A survey in these regions (MOH & WHO, 2015) revealed that complementary feeding diets were inadequate in terms of dietary quality, amount, and diversity to meet nutritional requirements. Timely complementary feeding and minimum meal frequency were practiced by just under half of the children between the ages of 6 and 23 months (48% and 47.8%, respectively). Just over ten percent (11.2%) consumed foods from the recommended minimum number of food groups considered necessary for a nutritionally adequate diet.

The WHO/UNICEF Global Strategy for Infant and Young Child Feeding recommends early initiation of breastfeeding, exclusive breastfeeding for 6 months, and the introduction of adequate complementary foods at 6 months with continued breastfeeding for 2 years or beyond (WHO & UNICEF, 2003). Although nearly all children are breastfed, after six months of age the energy and nutrient contribution from complementary food becomes increasingly important for meeting daily requirements. For most of them, however, the foods fed to them do not contain enough energy and micronutrients to meet daily requirements (Dewey & Brown, 2003; Gibson & Ferguson, 1998; Hotz & Gibson, 2007; Nestel et al., 2003). Meeting nutrient demands during complementary feeding is, however, challenging as the amount of nutrients needed to support the rapid growth and development that occurs at this age is significant (Brown, Dewey, & Allen, 1998; Dewey & Brown, 2003; Nestel et al., 2003; Victora et al., 2010). Filling the nutrient gaps not covered by breastfeeding is particularly difficult due to both the high nutrient requirements and nutrient density of foods. Nutrient dense foods are necessary to ensure that nutrient requirements are met without displacing breastfeeding (Dewey & Adu‐Afarwuah, 2008; Dewey & Brown, 2003; Hotz & Gibson, 2007). The WHO recommends the promotion of locally available and produced foods where possible, if they are able to address critical nutrient gaps (WHO, 2008). However, most developing countries lack context‐specific food‐based complementary feeding recommendations (Dewey & Brown, 2003).

Under the Accelerating of Nutrition Improvements (ANI) project (ANI, 2013), the WHO supported the Ministry of Health (MoH) in Uganda to develop context‐specific food‐based complementary feeding recipes (FBCFRs) for the eastern and western regions. These are the first of their kind to be developed in the country, and the approach was based on both *Pro*PAN and Optifood tools. ProPAN is a structured process that can be undertaken to improve the quality and coverage of IYCF programming. Optifood is a linear programming software tool that can be applied to provide an evidence base for the development of messages used to improve IYCF practices (Daelmans et al., 2013). *Pro*PAN and Optifood tools have previously been used to develop context‐specific food‐based complementary feeding recommendations in Kenya (Vossenaar et al., 2017); Myanmar (Hlaing et al., 2016); Cambodia (Skau et al., 2013); and Indonesia (Santika, Fahmida, & Ferguson, 2009).

An interface between *Pro*PAN and Optifood corresponds to the testing of the food‐based recommendations to assess their feasibility and acceptability for long‐term use by the target population prior to dissemination using trials of improved practices (TIPs), a qualitative approach (Daelmans et al., 2013). TIPs, developed by Manoff group, consist of a series of visits in which the interviewer and the participant analyze current practices, discuss what could be improved, and together reach an agreement on one or a few solutions to try over a trial period and then assess the trial experience together at the end of the trial period (Manoff‐Group, 2018).

When applied to IYCF, TIPs identify improved practices that are acceptable and feasible for families to implement (Dickin & Griffiths, 1997). As all practices are tested, ideally, in people's homes before they are recommended for use in a larger program, TIPs provide an opportunity to learn directly from program participants. Additionally, TIPs give families the chance to try a new behavior, while program planners/implementers learn from them about what is culturally feasible and acceptable (Dickin & Griffiths, 1997).

In Afghanistan, Zambia, Malawi, Laos, and Cambodia, TIPs have been successfully used to evaluate the feasibility and acceptability of complementary feeding practices among children (Wijesinha, Kennedy, Dirorimwe, & Muehlhoff, 2013). The use of TIPs to assess the feasibility and acceptability of context‐specific FBCFRs has not been done in Uganda. This paper describes the process of using TIPs to test the feasibility and acceptability of FBCFRs developed for eastern and western regions of Uganda. The results of this study are important in contributing to the understanding of facilitators and barriers to the acceptability and feasibility in using context‐specific FBCFRs in Uganda. This information provides valuable guidance for the implementation of complementary feeding programs in Uganda.

### 1.1 Food‐based complementary feeding recommendations

As part of the ANI project, FBCFRs were developed for the eastern and western regions of Uganda (Tables 1 and 2). Recommended to be used in addition to other family foods, the FBCFRs targeted the age groups 6–8, 9–11, and 12–23 months’ breast and nonbreastfed children. These recipes were intended to be promoted alongside other recommended principles of infant and young child feeding (WHO/PAHO, 2003).

**Table 1:** Food‐based complementary feeding recipes for the eastern region

Target group		Food‐based commentary feeding recipe	Frequency of consumption/week	Food item/Serving sizes	Frequency of meals/day	Additional information
6–8 months		1. Millet + milk	4	Millet−85 g Milk−55 g Mukene−2 g	2–3 times	Distribute mukene in food
		2. Millet + mukene	1			
		3. Swtpot. + beans + gnuts + GLV	1	Swt pot−45 g Beans−10 g Gnut−5 g Glv−10 g Mukene−2 g		
		4. Swtpot + Gnut + mukene + GLV	3			
		5. Swtpot + bean + mukene + GLV	3			
		6. Beans + Gnut	3			
9–11 months		1. Millet + milk	7	Milk−70 g Millet−80 g	Four times (3 meals plus 1 nutritious snack in between meals)	
		2. Swtpot. + beans + gnuts + GLV	3	Swtpot−60 g Beans−20 g Gnut−10 g GLV−15 g Mukene−10 g		
		3. Swtpot + Gnut + mukene + GLV	2			
		4. Swtpot + bean + mukene + GLV	2			
		5. Gnut + beans	2			
12–23 months (breastfeeding)		1. Millet + milk	7	Milk−100 g Millet−205 g	Five times (3 meals plus 2 nutritious snacks between meals)	
		2. Swtpot. + beans + gnuts + GLV	2	Swtpot−90 g Beans−40 g Gnut−10 g GLV−25 g Mukene−10 g		
		3. Swtpot + Gnut + mukene + GLV	2			
		4. Swtpot + bean + mukene + GLV	2			
		5. Gnut + beans	3			
12–23 months (nonbreastfeeding)		1. Millet + milk	7	Milk−80 g Millet−140 g Mukene−10 g	Five times (3 meals plus 2 nutritious snacks between meals)	Give an extra cup (250 ml) of milk twice every day
		2. Mukene + millet	1			
		3. Swtpot. + beans + gnuts + GLV	1	Swtpot−90 g Beans−40 g Gnut−10 g GLV−15 g Mukene−10 g		
		4. Swtpot + Gnut + mukene + GLV	3			
		5. Swtpot + bean + mukene + GLV	3			
		6. bean + gnut	3			

Note

Swtpot: sweet potato, gnut: groundnut, GLV: green leafy vegetables, “mukene” (silverfish)

**Table 2:** Food‐based complementary feeding recipes for the Western region

Target group	Food‐based commentary feeding recipe no.	Frequency of consumption/wk	Food item/Serving sizes	Frequency of meals/day	Additional information
6–8 months	1. Millet + milk	4	Millet−155 g Milk−50 g Soy flour−20 g Eggs−40 g	2–3 times	Give fruit as a snack (at least 35 g)
	2. Millet + eggs	1			
	3. Soy + milk	2			
	4. Gnuts + beans + GLV	4	Beans−20 g DGLV−10 g Gnuts−10 g		
	5. Beans + GLV	3			
9–11 months	1. Millet + milk	5	Millet−135 g Milk−80 g Soy flour−65 g Milk−80 g	Four times (3 meals plus 1 nutritious snack in between meals)	Give fruit as a snack (at least 55 g)
	2. Millet + eggs	1			
	3. Soy + milk	2			
	4. Gnuts + beans + GLV	3	Beans−30 g DGLV−10 g Gnuts−10 g		
	5. Gnuts + Eggs + GLV	1			
	6. Beans + GLV	4			
12–23 months (breastfeeding)	1. Millet + milk	4	Milk−115 g Eggs−45 g Millet−200 g Soy flour−55 g	Five times (3 meals plus 2 nutritious snacks between meals)	Give 2 boiled eggs once a week as snack
	2. Millet + eggs	1			
	3. Soy + milk	3			
	4. Gnuts + beans + GLV	4	Gnuts−15 g Beans−45 g GLV−45 g		Give fruit as a snack (at least 65 g)
	5. Beans + GLV	3			
	6. Gnuts + GLV	3			
12–23 months (nonbreastfeeding)	1. Millet + milk	4	Millet 115 Milk−90 g Eggs−62 Soy flour−177 g	Five times (3 meals plus 2 nutritious snacks between meals)	Give:‐a cup of milk daily −3 eggs
	2. Millet + eggs	1			
	3. Soy + milk	3			
	4. Gnuts + beans + GLV	4	GLV−50 g Beans−40 g Gnuts−15 g Millet−75 g		Give fruit as a snack every day (at least 70 g)
	5. Beans + GLV	3			
	6. Gnuts + GLV	3			

Note

GLV: green leafy vegetables; Gnut: groundnuts; soy: soybeans.

#### 1.1.1 Food‐based complementary feeding recommendations for the eastern region

In the eastern region, the FBCFRs covered foods that included seven foods: millet, milk, mukene (silverfish), sweet potatoes, beans, groundnuts, and green leafy vegetables. There was an additional recommendation of providing nonbreastfed children with at least 250 ml of milk, twice daily. The feeding frequency followed the WHO recommendation (WHO/PAHO, 2003) for the different age groups for IYCF where children 6–8 months are supposed to be fed 2–3 times a day and four times (three meals plus one nutritious snack) a day for children 9–11 months of age. Breast and nonbreastfed children 12–23 months of age had a recommended feeding frequency of five times. Similar to the younger age groups, the fifth meal was intended to be a nutritious snack (Table 1).

#### 1.1.2 Food‐based complementary feeding recommendations for the Western region

Like the eastern region, the FBCFRs in the western region covered seven foods including millet, milk, eggs, soy, groundnuts, beans, and green leafy vegetables. In addition, there were additional recommendations of providing a fruit as a snack for children across all age groups and eggs and milk for 12‐ to 23‐month‐old children (Table 2).

## 2 METHODS

### 2.1 Study area and sampling

The study was carried out between August and September 2015 in the eastern (Iganga, Luuka, and Namutumba districts) and western regions (Hoima, Masindi, and Kibaale districts) of Uganda. The two regions were part of the ANI project. A two‐stage purposive sampling approach was used to select participants to take part in the TIPs exercise that used both qualitative and quantitative methods. Purposive sampling was used to select participants for the focus group discussions (FGDs) to ensure they had similar characteristics. First, from each of the districts, a subcounty and village were selected randomly. Village Heath Teams from each of the selected communities were then asked to contact and select caretakers with children between the ages of 6–8 months, 9–11 months, 12–23 months (breastfeeding), and 12–23 months (nonbreastfeeding). Key informant interview (KII) participants were selected on the basis of them being in influential positions in the community. The final sample size, shown in Table 3, comprised of 60 TIPs, 60 FDG, and 3 KIIs in each region. All participants provided individual informed consent to participate. The study protocols were approved by the Uganda National Council of Science and Technology.

**Table 3:** Sample size of respondents per region

Data collection method	Eastern Region						Western Region					
	Mothers of children				Fathers of children	Key Informants	Mothers of children				Fathers of children	Key informants
	6–11 months	9–11 months	12–23 months breastfed	12–23 months nonbreasted	6–23 months		6–11 months	9–11 months	12–23 months breastfed	12–23 months nonbreasted	6–23 months	
Baseline TIPs questionnaire	19	11	15	15			17	13	15	15		
TIPs household visit 1	19	11	15	15			17	13	15	15		
TIPs household visit 2	19	11	15	15			17	13	15	15		
TIPs household visit 3	19	11	15	15			17	13	15	15		
FGDs with mothers	3 (x 10 participants)					3 (x 10 participants)						
FGD with fathers					3 (x 10 participants)						3 (x 10 participants	
Key informant interviews						3						3
Total data collection	60 Baseline questionnaires 180 TIPs Interviews 6 FGDs 3 x KIIs						60 Baseline questionnaires 180 TIPs interviews 6 FGDs 3 x KIIs					
Total sample size	60 TIPs participants 60 FGD participants 3 x KII participants						60 TIPs participants 60 FGD participants 3 x KII participants					

### 2.2 Data collection

Before the start of each household visits, trained research assistants explained the purpose of the study to the caretakers and requested them to participate in data collection at four time points including an initial that included food demonstration to allow the caretakers participate in preparation of different recipes that were being promoted. This was followed by three household visits of trials of improved practices (TIPs), the standard approach to implementation.

#### 2.2.1 Food demonstration

Data collection to assess the feasibility and acceptability of the recipes started with a food demonstration where community members were taught how to prepare the FBCFRs. Issues such as the recommended consistency of porridges and how to feed it to the children were emphasized, among others. After this exercise, actual data were collected over a 4‐week period as described under the sections below.

#### 2.2.2 Household visit 1

Household visit 1 assessed the socio‐demographic factors using a semistructured questionnaire. A food frequency questionnaire was also administered to understand the dietary intake of the participating children and their household. In addition, following techniques for discussing the new feeding practices as outlined in the TIPs and ProPAN guides, participating families were introduced to the FBCFRs for their relevant target group and asked to try them for a period of 3 weeks.

#### 2.2.3 Household visit 2

A second household visit, conducted after a 7‐day period from the first one, assessed the actual use, barriers, and difficulties related to using the FBCFRs introduced at household visit 1 using a semistructured questionnaire. Any changes and substitute foods that have been introduced were also captured.

#### 2.2.4 Household visit 3

The third and final household visit took place 22 days after the first visit. Using a semistructured questionnaire, this visit assessed the participants’ attempts to practice the FBCFRs and if they intended to continue using them. Any barriers and facilitators to their continued use were also explored. In addition, a food frequency questionnaire was used to assess dietary intake and patterns of the participating children and their household.

#### 2.2.5 FGDs with the mothers/caretakers of children 6–23 months

Using a structured guide, focus group discussions were carried out with mothers/caretakers of children 6–23 months of age to understand the insights into community norms, which can be used as a framework to help explain the variation in the infant and young child feeding (IYCF) practices of households participating in the FBCFRs trials. In each region, there were three focus group discussions of 10 participants each. Key information sought from the focus group discussions included participants’ perceptions about feeding young children, the challenges that they faced in feeding children, reactions to the FBCFRs, and other issues of IYCF. All focus group discussions were conducted out in the local language and were audiotaped.

#### 2.2.6 Focus groups with Fathers of children 6–23 months

Focus group discussions in each region explored the feasibility and acceptability of the FBCFRs with fathers, using a structured guide. Involving 10 participants each, each region held three separate focus group discussions. The discussions included the feasibility of putting the FBRs into practice with reference to production, cost, availability, and seasonality of the promoted food items. Similar to caretakers, FGDs with fathers were also conducted out in the local language and were audiotaped.

#### 2.2.7 Key informant interviews

Three key informant interviews, per region, with selected community leaders took place to discuss insights on the choices that people are making and current behaviors on IYCF in the community. Because food production usually determines availability at household level and hence practicing the FBCFRs, issues around agricultural production were also explored. Interviews focused on themes about which the selected informants had specialized knowledge in. These interviews took place in the local language and were audiotaped.

#### 2.2.8 Data Quality

For each region, the TIPs exercise collected data from different sources through the household visits, focus group discussion, and key informant interviews to allow for triangulation and corroboration of findings. All research assistants were experienced in qualitative and dietary assessments. Information on the practice of FBCFRs for individual children was cross‐checked with the reported data from the food frequency questionnaire. At the end of each field visit, data checking to ensure consistency was done. Where irregularities arose, they were discussed by researchers.

### 2.3 Data analysis

Background characteristics of the respondents and data on food frequency were analyzed using SPSS version 19 and summarized as frequencies. Data from qualitative interviews were transcribed verbatim. All interviews were translated into English during the transcription process. During transcribing, the question was written in bold and the response in normal text. This format facilitated distinguishing between the interviewer and respondent. After careful reading and re‐reading of the transcripts together with re‐listening to the audio tapes, coding schemes of relevant emerging themes related to acceptability and feasibility of putting the FBCFRs into practice were developed using the grounded theory technique in which each strand of information leads to further investigation of behaviors and activities in the data to identify common themes (Strauss & Corbin, 1990). Code development was both literature and data driven and was done by both authors to make sure the identified themes matched the data.

In addition to the thematic analysis, mothers’/caretakers’ responses were categorized as frequencies by indicators of acceptability and feasibility of the practices. This included willingness to practice the recipes, actual practicing of the recipes, any modifications made, and intention to continue using the recipes.

## 3 RESULTS

### 3.1 Socio‐demographic characteristics of participants

Table 4 shows the demographic characteristics of the surveyed mothers and their children. In both regions, 6 out of every 10 children in the sample were male. Similarly, nearly six out of every ten mothers/caretakers in both regions were in the age bracket of 20–29 years of age. Compared to four out of every ten caretakers in the western region, six out of every ten had not completed primary school level in the eastern region. Consequently, as many as seven out of every ten (76.7%) compared to only four out of every ten (40%) caretakers had the ability to read and write in the western compare to the eastern region, respectively. Respondents from the eastern region also reportedly had more other children in the household compared to their western counterparts. Twenty‐eight percent, in the eastern region compared to 8.3% in the western, of caretakers were in polygamous marriages.

**Table 4:** Socio‐demographic characteristics of surveyed children and their caregivers

Variable	Eastern region (*n* = 60)	Western region (*n* = 60)
	No (%)	No (%)
Age of child		
6–8 months	19 (31.7)	17 (28.3)
9–11 months	11 (18.3)	13 (21.7)
Breastfed children aged 12–23 months	15 (25)	15 (25)
Nonbreastfed children 12–23 months	15 (25)	15 (25)
Sex of child		
Male	37 (61.7)	36 (60.0)
Female	23(38.3)	24 (40.0)
Mother/Caretaker age		
<20	5 (8.3)	7 (11.7)
20–29	38 (63.3)	34 (56.7)
30–39	16 (26.7)	17 (28.3)
=>40	1 (1.7)	2 (3.3)
Language		
Lusoga	41(68.3)	0 (0.0)
Lusiki	19 (31.7)	0 (0.0)
Runyoro	0	51 (85.0)
Luganda	0	3 (5.0)
Runyankore	0	6 (10.0)
Level of education		
No formal education	9 (15)	9 (15.0)
Incomplete primary	37 (61.7)	24 (40.0)
Completed primary	3 (5.0)	10 (16.7)
Secondary (S1–S4)	10 (16.7)	16 (26.7)
Secondary (S1–S4)	1 (1.6)	1 (1.6)
Ability to read and write		
Yes	25 (41.7)	46 (76.7)
No	35 (58.3)	14 (23.3)
No. other children <5 years in HH		
1	11 (18.3)	24 (40.0)
2–3	45 (75.0)	34 (56.7)
>3	4 (6.7)	2 (3.3)
Marital status		
Single	3 (5.0)	8 (13.4)
Monogamous marriage	39 (65.0)	43 (71.6)
Polygamous marriage	17 (28.3)	5 (8.3)
Divorced	1 (1.7)	4 (6.7)

#### 3.1.1 Socio economic status of surveyed households

In both regions, nearly all mothers/caretakers were not employed. The few who were employed (9% and 12% for eastern and western regions, respectively) were all self‐employed (data not shown).

#### 3.1.2 Decision making about purchase of food in a home

Almost all mothers/caretakers (80%) in both regions mentioned that their partners or husbands are the main decision makers when it comes to purchase of food in the households (data not shown).

#### 3.1.3 Source of food in households

**Figure 1:**
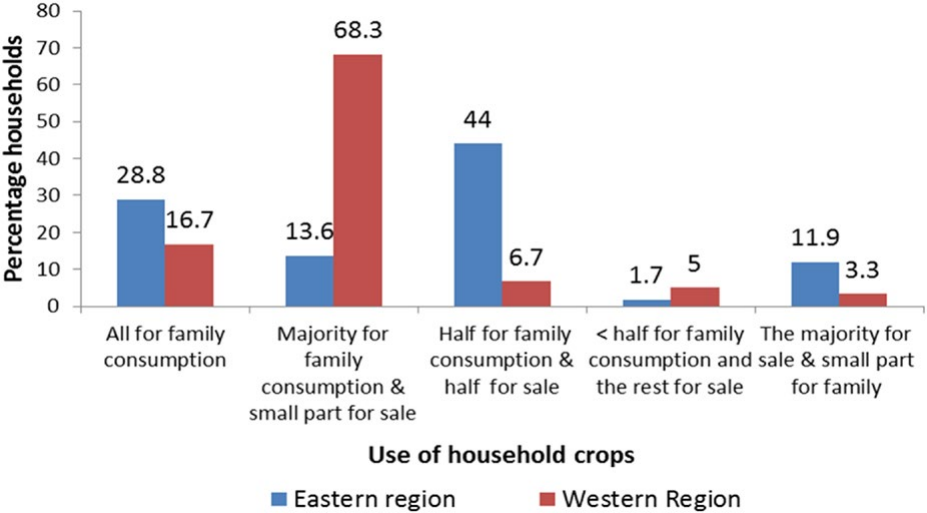
Use of household food crops in eastern versus western regions

Majority of the households in the western and eastern regions produce their own food. The majority of households in western Uganda use a small part for sale and the larger portion for home consumption. On the contrary, households in eastern Uganda sell half of the food (Figure 1).

### 3.2 Feasibility of complementary feeding recipes in Eastern Uganda

**Figure 2:**
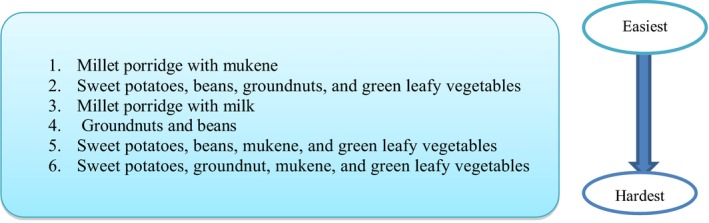
Scale of difficulty in implementing food‐based complementary feeding recommendations in eastern Uganda

Feasibility was defined as the ease with which a specific recipe was put into practice or implemented. The recipes of millet porridge and mukene and sweet potatoes, beans, groundnuts, and green leafy vegetables were the easiest to implement. The recipes containing millet porridge with milk and those where mukene was added to food were the hardest to implement (Figure 2). Overall mothers, however, found it difficult to implement a full set of recipes together. In addition, the recommended frequencies were not adhered to across all target groups (data not shown).

### 3.3 Acceptability of recipes in the Eastern region

For each target group, acceptability of recipes was defined by the proportion of caretakers that tried/practiced/implemented the recipes, with or without modifications (Table 5). Nonacceptable was defined by having none of the mothers try a given recipe.

**Table 5:** Acceptability of recipes in the Eastern region

Target group	Food‐based commentary feeding recipe no.	Tried recipe at visit 1 no. (%)	Tried recipe at visit 2 no. (%)	Modification/replacements made (no. mothers)	Intend to continue using no. (%)
6–8 months	1. Millet + milk (*n* = 19)	19 (100)	19 (100)	None	16 (84)
	2. Millet + mukene (*n* = 19)	14 (73.5)	12 (63)	Millet with maize (3)	14 (73.5)
	3. Swtpot. + beans + Gnuts + GLV (*n* = 19)	15 (80)	12 (63)	None	15 (80)
	4. Swtpotatoes + Gnut + mukene + GLV (*n* = 19)	15 (80)	12 (63)	Sweet potato with maize (4) or millet (2)	12 (63)
	5. Swtpot + bean + mukene + GLV (*n* = 19)	13 (68.4)	10 (53)	Sweet potatoes with maize (4) or millet (3)	10 (53)
	6. Beans + gnut (*n* = 19)	12 (53)	12(63)	None	12 (63)
9–11 months	1. Millet + milk (*n* = 11)	11(100)	7 (64)	None	7 (64)
	2. Swtpot. + beans + gnuts + GLV (*n* = 11)	11(100)	11(100)	Sweet potato with millet (2), maize (3) and cassava (2)	11(100)
	3. Swtpot + Gnut + mukene + GLV (*n* = 11)	9 (82)	9(82)	None	9 (100)
	4. Swtpot + bean + mukene + GLV (*n* = 11)	7 (64)	7(64)	Sweet potato with maize (2)	7 (64)
	5. Gnut + beans (*n* = 11)	11(100)	6 (40)	None	6 (40)
12–23 months breastfeeding	1. Millet + milk (15)	15 (100)	11 (73)	None	11 (73)
	2. Swtpot. + beans + gnuts + GLV (15)	14 (93)	11(73)	Sweet potatoes with millet (2), maize (3) and cassava (1)	14 (93)
	3. Swtpot + Gnut + mukene + GLV (15)	13 (87)	10 (67)	None	10 (67)
	4. Swtpot + bean + mukene + GLV (15)	12 (80)	11 (73)	None	11 (73)
	5. Gnut + beans (15)	12 (100)	12 (100)	None	12 (100)
12–23 months nonbreastfeeding	1. Millet + milk (15)	13 (87)	10 (67)	Millet with maize (2)	9 (60)
	2. Mukene + millet (15)	13 (87)	8 (53)	None	12 (80)
	3. Swtpot. + beans + gnuts + GLV (*n* = 15)	12 (80)	11 (73)	Sweet potatoes with maize (3)	11 (73)
	4. Swtpot + Gnut + mukene + GLV (*n* = 15)	11 (73)	10 (67)	Sweet potatoes with maize (2)	9 (60)
	5. Swtpot + bean + mukene + GLV(*n* = 15)	13 (87)	10 (67)	None	13 (87)
	6. Beans + gnut (*n* = 15)	10 (67)	8 (53)	None	8 (53)

Note

GLV: green leafy vegetables; Gnuts: groundnuts; Swtpot: sweet potatoes.

For all target groups, all the recipes in the eastern region were acceptable. Among the target group 6–8 months old, millet with milk recipe was accepted by 100% of all the caretakers, without any modifications. Over 80% of caretakers reported they would continue using it after the TIPs exercise. Generally, the recipes containing sweet potatoes, bean, groundnuts, and green leafy vegetables were the least acceptable, followed by those with millet. The least tried recipe was that containing Swtpot. + beans + mukene + GLV (53%). Among this age group, millet and sweet potatoes were commonly substituted with maize.

For children 9–11 months, millet plus milk and sweet potatoes, beans, groundnuts, and green leafy vegetables were the most acceptable recipes at visit 1. Sweet potatoes, beans, groundnuts, and green leafy vegetables (with or without mukene) were the recipes tried by all caretakers of children in this target group and accepted to continue using after TIPs. Similar to the target group 6‐ to 8‐month‐old children, the commonest substitution in this recipe was sweet potato for maize. The recipe least likely to be continued was beans and groundnuts as only 40% tried the recipe at visit 2 with the intention to continue.

An average of ninety‐two percent of mothers of breastfed children aged 12–23 months of age accepted all the recipes at visit 1. However, only 77% of  them tried at visit 2, and 81% intending to continue use. Contrary to the younger age groups, the gnuts + beans recipe was the most acceptable recipe and all caretakers in this age group intended to continue using it. Like other target groups, sweet potatoes were substituted with maize, millet, or cassava. The least likely recipe to be continued was sweet potato + groundnuts + mukene + green leafy vegetables.

Similar to breastfed children 12–23 months of age, over 90% of caretakers with nonbreastfed children tried the recipes at visit 1. Caretakers who tried the recipes at visit 2 (76%) and mentioned that they would continue using them (83%) were also within the same range. Like other target groups, sweet potatoes were substituted with maize, millet, or cassava.

### 3.4 Food frequency of food items in recipes before and after the implementation of TIPs

**Figure 3:**
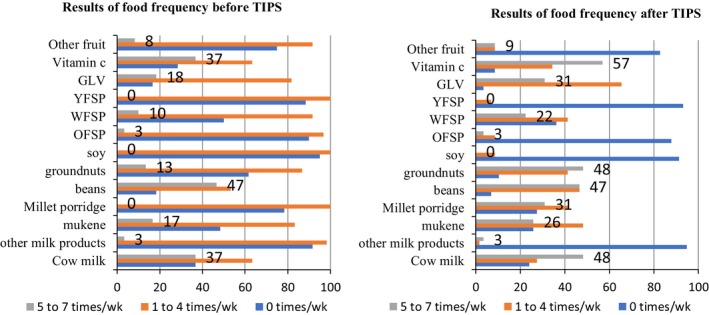
Food frequency of foods in the eastern region among children before and after TIPs exercise

The information in Figure 3 below shows that the consumption of most foods mentioned in the recipes for the eastern region improved after the TIPs exercise. The graphs show that the number of respondents who mentioned a child not eating a food item (zero times per week) lowered for all foods, except yellow‐fleshed sweet potato, orange‐fleshed sweet potato, and soya after the TIPs exercise. Similarly, the number of respondents for the food frequency of 5–7 times per week increased for all foods, except for groundnuts, yellow‐fleshed sweet potato, orange‐fleshed sweet potato, and soya. On the contrary, the food frequency for 1–4 times per week lowered after the TIPs exercise.

#### 3.4.1 Barriers and facilitators to feasibility and acceptability of recipes in Eastern Uganda

While the recipes were found to be generally acceptable, there were some barriers to their use that were noted such as money for ingredients that were not either available in season or needed purchase such as millet, milk, sweet potatoes, and groundnuts:

We do not grow millet and it is very expensive to buy. We also we do not have cows and thus have to buy milk which is expensive. (Mother’s FGD, Luuka)

I cannot guarantee practicing the recipe in this month because sweet potatoes are out of season; I promise to put into practice next month when I harvest my sweet potatoes. (Mother in Luuka)

The barrier I am ‘seeing’ is the one of groundnuts because they are expensive and some people may not have the money to afford them while those who grow them sell all of them. (Key Informant, Luuka)

Ingredients such as millet were more expensive when they were not in season:

We plant millet in in January and February and we start harvest in June and July, I think majority of households may have some millet to make the porridge during the trial period. However, when millet is not in season it is expensive very few households can afford. (Father’s FGD, Namutumba)

Millet is plenty in June to Feb and scarce in March to June and its scarcity in March to June makes it expensive. (Women’s FGD, Iganga)

Unavailability of some ingredients made others resort to preparing recipes with some modifications:

I may get money to buy milk or millet but not both yet I cannot steal the other so I will practice what I can afford. (Father’s FGD, Namutumba)

We should include maize instead of sweet potatoes because we use it (Maize) a lot in the community unlike sweet potatoes. (Key informant, Luuka)

Some caretakers had cultural beliefs associated with some foods in the recipes but this was only from Namutumba district:

I cannot give my child mukene or any other fish because we are ‘baswezi’ and we don’t eat it; it is regarded as a taboo; my husband can even leave me when he finds out I have given his children fish. (Mother in Namutumba)

Factors that affected acceptability of recipes also varied and included things such as nutritious, tasty, and neighbors’ willingness to test them:

I talked to my neighbour; she said she would try the recipe because it is tasty and when she gave it to the child, she liked. (Mother in Iganga)

Ever since I started giving my child this porridge with milk she plays a lot and has a lot of energy. (Mother in Iganga)

I am very committed to do anything, so long as it benefits my children. (Father in Luuka)

For some mothers, the motivation was from the small amounts of ingredients needed to make the recipes:

…..since it is only small amounts of millet and milk needed to make my child’s porridge, I will do it. (Mother in Iganga)

Similarly, key informants agreed with the mother's motivation of putting the recipe into practice because of the small quantities of ingredients.

The amounts recommended are very small and thus mothers will be able to put these recommendations into practice. (Key Informant, Iganga)

Mukene is usually purchased but since small amounts are needed mothers will be able to put this recipe into practice. (Key Informant, Iganga)

### 3.5 Feasibility of complementary feeding recipes in Western Uganda

**Figure 4:**
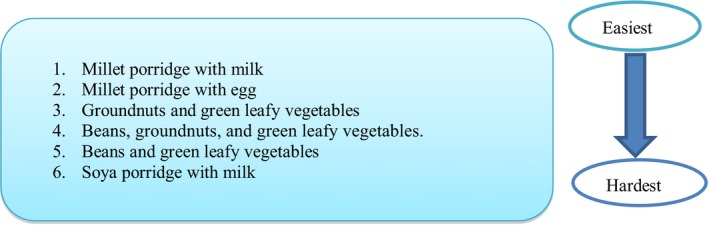
Scale of difficulty in implementing food‐based complementary feeding recommendations in western Uganda

Like the eastern region, it was not possible to put the complete set of recipes into practice as recommended across all the target groups. The recipes of feeding children on millet porridge and milk, and millet porridge with egg were more achievable overall than those for soya porridge and boiled milk, as shown in Figure 4.

### 3.6 Acceptability of recipes in the Western region

Like the eastern region, acceptability of recipes was defined by the proportion of caretakers that tried/practiced/implemented the recipes, with or without modifications (Table 6). Nonacceptable was defined by having mothers try a given recipe.

**Table 6:** Acceptability of recipes in the Western region

Target group	Food‐based commentary feeding recipe no.	Willing to try recipe at visit 1 no. (%)	Tried recipe at visit 2 no. (%)	Modification/replacements made (no. mothers)	Intention to continue use/acceptability no. (%)
6–8 months	1. Millet + milk (*n* = 17)	15 (88)	10 (71)	Millet with maize (5)	15 (88)
	2. Millet + eggs (*n* = 17)	12 (71)	12 (71)	None	12 (71)
	3. Soy + milk (*n* = 17)	5 (29)	5 (29)	None	5 (29)
	4. Gnuts + beans + GLV (*n* = 17)	17 (100)	17 (100)	None	17 (100)
	5. Beans + GLV (*n* = 17)	16 (94)	16 (94)	None	16 (94)
9–11 months	1. Millet + milk (*n* = 13)	13 (100)	13 (100)	Millet with maize (1)	13 (100)
	2. Millet + eggs (*n* = 13)	11 (85)	10 (77)	Millet with maize (1)	10 (77)
	3 .Soy + milk (*n* = 13)	2 (15)	2 (15)	None	0 (0)
	4. Gnuts + beans + GLV (*n* = 13)	12 (92)	11 (85)	None	11 (85)
	5. Beans + GLV (*n* = 13)	13 (100)	13 (100)	None	13 (100)
12–23 months Breastfed	1. Millet + milk (*n* = 15)	14 (93)	7 (47)	None	13 (87)
	2. Millet + eggs (*n* = 15)	9 (60)	8 (53)	None	8 (53)
	3. Soy + milk (*n* = 15)	1 (7)	1 (7)	None	1 (7)
	4. Gnuts + beans + GLV (*n* = 15)	12 (80)	12 (80)	None	12 (80)
	5. Beans + GLV (*n* = 15)	11 (73)	11 (73)	None	11 (73)
	6. Gnuts + GLV (*n* = 15)	15 (100)	15	None	15 (100)
12–23 months Nonbreastfed	1. Millet + milk (*n* = 15)	15 (100)	15 (100)	None	13 (87)
	2. Millet + eggs (*n* = 15)	12 (80)	12 (80)	None	8 (53)
	3. Soy + milk (*n* = 15)	0 (0)	0 (0)	None	0 (0)
	4. Gnuts + beans + GLV (*n* = 15)	14 (93)	14 (93)	None	14 (93)
	5. Beans + GLV (*n* = 15)	12 (80)	12 (80)	None	12 (80)
	6. Gnuts + GLV (*n* = 15)	12 (80)	12 (80)	None	12 (80)

Note

Gnuts: groundnuts; GLV: green leafy vegetables.

Among the target group 6‐ to 8‐month‐old children, millet plus milk and milk plus egg were acceptable and tried by 8 (88%) and 7 (71%) out every ten mothers, respectively. However, some mothers replaced millet with maize as a modification. Beans and groundnuts recipes with or without green leafy vegetables were acceptable and tried by over nine out of ten mothers (94%). The list likely recipe to be continued was for this age group was soy with milk (29%).

Similar to the target group 6–8 months old, all recipes were accepted and tried on average by 8 out of 10 mothers except for soy and milk where only 1 out of 10 mothers among the 9–11 months’ target group but with some mothers replacing millet with maize. Beans + gnut recipes with or without green leafy vegetables were acceptable by over 85% of caretakers. Millet was replaced with maize in this target group.

Whereas there were no modifications for this target group, the results for the target group 12–23 months breastfed children were not different from the 9–11 months’ target group for all recipes, except the millet and egg—only 7% caretakers accepted this recipe. Only 6 out of every 10 caretakers tried the recipes at visit 2 and were willing to continue using except for soy and milk and millet and eggs.

Similar to the 12–23 months breastfed children, nonbreastfed children had no modifications in recipes. Over 80% of caretakers for children in the target group 12–23 months of age nonbreastfed children accepted most recipes (except for the soy + milk) with the intention to continue use except for millet and milk and eggs and soy and milk

### 3.7 Food frequency of foods in the Western Region among children before and after TIPs exercise

**Figure 5:**
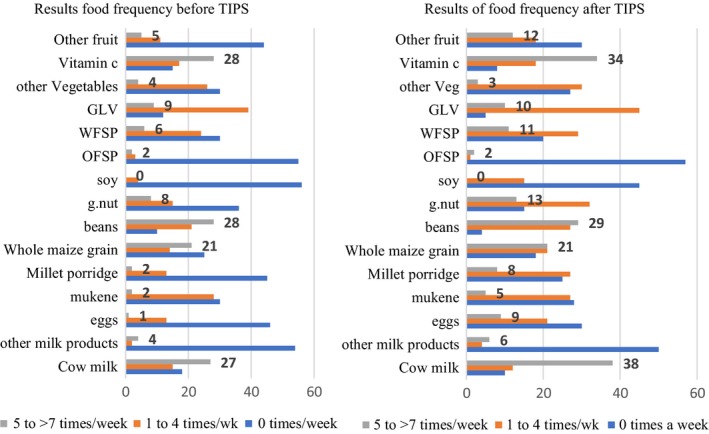
Food frequency of foods in the western region among children before and after TIPs exercise

The information in Figure 5 shows that the consumption of most foods mentioned in the recipes for the western region improved before and after the TIPs exercise. The graphs show that the number of respondents who mentioned not eating a particular food item (zero times per week) lowered for all foods, except for other vegetables orange‐fleshed sweet potato, after the TIPs exercise. Similarly, the number of respondents for the food frequency 5–7 times per week increased for all foods except other vegetables, orange flesh sweet potato, soy, and whole maize grain. On the contrary, the food frequency for 1–4 times per week lowered after the TIPs exercise with proportional increase in food frequency of 5–7 times.

### 3.8 Barriers and facilitators to feasibility and acceptability of recipes in Western region

Soy porridge with milk was the hardest recipe to implement because soya is grown by a few people and costly in the region as was mentioned by some participants below:

Soya is not commonly grown and yet very expensive in the market. (Mother FGD Kibaale)

I also think that the recipe (soy + millet) will be hard to follow because some foods such as soya are rare in the community. (KI Masindi)

One of the barriers for not growing soya was the lack of market. This was cited by one of the male participants:

Soya is not commonly grown because there is no market for it. (Father’s FGD, Hoima)

Similar to soy + milk recipe, there were concerns of the cost of eggs and milk needed to implement the millet plus egg and millet and milk recipes as shown below:

I need money to buy eggs to be able to practice this recipe (millet + eggs). (Mother FGD Masindi)

Milk is expensive during the dry season because it is available in less quantity in this community. (Mother FGD, Hoima)

For some mothers, in addition to cost, milk was not in the neighborhood:

Milk, is also bought from a market which is about 2–4 miles we need money and means of transport‐motorcycle, bicycle to get there. (Mothers FGD, Hoima)

It was also noted that milk had to be bought in the morning because of its high demand yet the supply is low. This was cited from FGD in Masindi:

Milk is always bought early or need booking before, otherwise, you don’t find any milk. (Mother in Masindi)

For some mothers, however, they were not very conversant with some recipes such as the millet plus egg one.

I still need more training to learn how to make this recipe (millet plus egg); I always do not do it right at the point of adding the egg and mixing. (Mother FGD Hoima)

However, just like other foods not grown by the households, mothers mentioned that they needed financial support from their partners. This was confirmed by a key informant in Masindi:

Women need money from their husbands to buy the milk since they do not have cows in their households. (Key informant Masindi)

It was noted that women need their partner's financial and social support in implementing this recipe. This was cited by one of the key informants:

Men are the most important group to involve in such health and nutrition meetings, with information, they can easily do some of the practices like ensuring that milk is available or even feeding the children themselves. (Key informant, Masindi)

Availability of the food was a key facilitator for practicing the recipes.

According to me the beans and greens are the common ones we get from our area. (Fathers FGD, Masindi)

……the issue concerning seasonality of groundnuts……green leafy vegetables are not usually grown and are scarce during the dry season…...( key informant interview Hoima)

According to me the beans and greens are the common ones we get from our area. (Fathers’ FGD, Masindi)

The ease of cooking and the cooking time were also key in acceptability of recipes:

This recipe (millet porridge plus egg) is good because within a few minutes after making the porridge, you just add the egg mix and serve your child. (Mother FGD Kibaale)

This recipe (millet porridge plus egg) is good because within a few minutes after making the porridge, you just add the egg mix and serve your child. (Mother FGD Kibaale)

To some mothers, child preferences made it easier for them to accept recipes:

I think my child likes the porridge (millet porridge with milk) because he keeps asking for more. (FGD Mother in Hoima)

We usually fry the eggs using cooking oil and the children like these eggs very much…….. (Father’s FGD, Kibaale)

Because the porridge recipes emphasized the need for thickness, some mothers testified that thick porridge made their children satisfied, grow, and gain more energy. This was confirmed by one of the mothers who stated that:

Ever since I started feeding my child on thick millet porridge mixed with milk she does not get hungry and plays a lot. (Mother FGD Masindi)

Specifically, to fruits, caretakers mentioned that apart from seasonality, children enjoyed the fruits and they would continue providing these.

…..fruits such as passion fruits are eaten by most families…….the children liked eating them….. (Mother FDG Masindi)

It was also found out that health workers and other family members were already promoting fruit consumption for better health:

Some of these foods such as mangoes and passion fruits have been recommended by our health workers to feed them to our children; therefore, we can even prepare what we have learned in our homes. (Mother’s FGD)

My mother in law has told me that fruits are good for my child and it prevents illnesses. (Mother in Kibaale)

## 4 DISCUSSION

Optimal complementary feeding practices for infants and young children in low‐income countries are a global public health concern because of its role in growth, development, and well‐being (WHO & UNICEF, 2003). While most infants and young children are breastfeed, complementary feeding practices are usually inadequate and as a result, most of them suffer from growth faltering during this time (Victora et al., 2008). Improving and optimizing diets of infants and young children is, therefore, essential to reduce not only the associated undernutrition, but also the increased risk of morbidity and mortality during this time (Bhutta et al., 2008; Jones et al., 2003). The WHO recommends the promotion of locally available and produced foods where possible, if they are able to address critical nutrient gaps during complementary feeding (WHO & UNICEF, 2003; WHO, 2008).

Under the ANI project (ANI, 2013), the WHO supported the Ministry of Health in Uganda to develop context‐specific food‐based complementary feeding recipes (FBCFRs) for use in the eastern and western regions of the country (Minstry of Health, 2015). The approach was based on the recommended methods of *Pro*PAN and Optifood (Daelmans et al., 2013).* Pro*PAN and Optifood have been used to develop complementary feeding in several other countries where complementary feeding practices are inadequate (Ferguson et al., 2006; Hlaing et al., 2016; Santika et al., 2009; Skau et al., 2013; Vossenaar et al., 2017). Trials of improved practices (Manoff‐Group, 2018), a standard approach, were used to test the feasibility and acceptability of the FBCFRs.

Although many studies have developed FBCFRs, to the best of our knowledge, feasibility and acceptability have only been reported from Myanmar (Hlaing et al., 2016), and FAO supported programs in Afghanistan, Zambia, Laos, and Cambodia (Wijesinha et al., 2013). Similar to the findings from these studies (Hlaing et al., 2016; Wijesinha et al., 2013), the findings from this study indicate that FBCFRs are feasible and acceptable, an important factor that promotes sustainability. Contrary to others (Hlaing et al., 2016) who explained that feasibility and acceptability were dependent on caretakers ability to overcome beliefs about certain foods, findings from this study showed that the FBCFRs were feasible and acceptable because of the familiarity of the foods to the caretakers. It is important to note that the degree of acceptability and feasibility, however, varied among families in this study when the food item in the recipe was not available in that season, (e.g., soy was not in season in western Uganda) as was also reported from Afghanistan, Zambia, and Cambodia (Wijesinha et al., 2013). This emphasizes the point that local availability and seasonality are key in determining feasibility and acceptability of the FBCFRs. In addition, the FBCFRs with familiar methods of preparation and ingredients remained the most tried and acceptable in both regions. For example, FBCFRs that included millet porridge with milk, groundnuts with green leafy vegetables, or beans in western Uganda were most acceptable showing that mothers easily accept to work with familiar foods and preparation methods.

While seasonality of foods can be solved by economic access, this may not work for low‐income families (Wijesinha et al., 2013) further risking malnutrition. It has been explained that during seasonality, children may be severely affected if food is preferentially allocated to the more productive members of the household (who are usually adults). It has also been argued (Wijesinha et al., 2013) that as a coping strategy, some mothers may intentionally decrease their personal food intake in order to protect the nutritional status of their children. While this coping mechanism may protect the nutritional status of the child, pregnant and lactating mothers are nutritionally vulnerable due to the increased nutrient needs (FAO & WHO, 2005; WHO & UNU, 2007). Some have suggested increasing incomes and possibly subsidizing the cost of certain foods, or supporting agricultural production as an approach to enabling poor families to have better quality diet in a sustainable manner but the evidence to support improved nutritional status this is weak (Vollmer et al., 2014). Increasing access to key food items could achieve a dual goal of diversifying diets with nutritious foods as well as increasing access to local products (Wijesinha et al., 2013).

On the one hand, families can be encouraged to substitute foods that are not in season. This, hence, means that FBCFRs need to provide alternative substitutes to provide the same nutrient requirements to take into account seasonal variation. Although mothers substituted foods in this study, it is possible that the nutrient quantities of the substituted foods did not match adequate nutrient intake, especially of the limiting nutrients. This, however, should not be a problem as the messaging behind the FBCFRs emphasized that families should use these in addition to other family foods. We, however, believe that proper use of the FBCFRs should improve the quality of complementary feeding among the children in the study because of the diverse foods in the recommendations.

Contrary to what others observed (Hlaing et al., 2016), cultural practices did not affect the acceptability and use of the recipes apart from a minority group from Namutumba district, in the eastern region. This could largely be attributed to the IYCF counseling services that were being promoted as part of the ANI project. This further emphasizes that behavior change may be easier when recipes are made from familiar foods.

A major limitation that was noted in this study is the adherence to the recommended feeding frequencies of the different recommendations, a similar finding elsewhere (Hlaing et al., 2016). In such low‐income settings, frequency of feeding among caretakers has been limited by some factors such as mothers time as was also found in this study. While in most African settings care for children is traditionally a mothers’ role, programs are introducing a gender dimension to this and there may be possibilities that families look at care for children as shared responsibility. In addition to mothers’ time, others (Wijesinha et al., 2013) explained that frequency of feeding can also be limited by availability of fuel for cooking as was found in this study. Caretakers reported that foods such as beans consumed more fuel and were a barrier to frequent feeding. Sensitizing communities on cooking methods such as soaking is key in conserving fuel. Moreover, soaking also reduces inhibitors such as trypsin, further increasing the quality of the diet (Hotz & Gibson, 2007). In this study, not adhering to the recommended feeding frequencies could also mean that some barriers to changing practices persisted as evidenced by the findings from the food frequency. Nevertheless, the results point to some specific foods that should be further explored for improved frequency of consumption.

## 5 CONCLUSION

Through TIPs, mothers demonstrated that they are open to try new ways of improving their children's nutrition. These context‐tailored recommendations can be implemented, with substitution of ingredients with similar nutrient values, in areas with similar foods. However, issues of sustainability and scalability need to be explored.

## ETHICAL APPROVAL

Ethical approval was obtained from Mildmay Human Ethics Review Committee, and thereafter, the study protocol was registered by the Uganda National Council of Science and Technology. Participants were informed about the study procedures before they gave informed consent. To safeguard confidentiality of information collected, respondents were identified by code numbers, permission of unauthorized persons to access completed questionnaires was not allowed, completed forms were stored securely, and all field interviewers were not allowed to discuss the respondents completed recalls with anyone except the field supervisor.

## CONFLICT OF INTEREST

The authors have no conflict of interest.

General Disclaimer: The named authors alone are responsible for the views expressed in this publication. In no event shall WHO be responsible for the content of this publication.
